# Texas State Medical Association

**Published:** 1893-05

**Authors:** 


### Society Notes.

### TEXAS STATE MEDICAL ASSOCIATION.

The twenty-fifth annual session of the Texas State Medical Association was held in Galveston, Texas, May 2d to 5th inclusive, Dr. J. D. Osborn, of Cleburne, President, in the chair, Dr. H. A. West, of Galveston, Secretary.

### MINUTES OF THE MEETING.

Association called to order at 11 a. m. Tuesday, May 2d inst., in Harmony Hall, by H. A. West, M. D., chairman Committee of Arrangements. Rev. Dr. Scott opened the session with prayer, invoking the Divine blessing on the deliberations of the meeting, and asking for Divine guidance in the search for truth.

Hon. R. L. Fulton, Mayor of Galveston, was then introduced, who tendered "the freedom of the city" to the doctors, in a neat and facetious address of welcome.

Dr. George H. Lee, of Galveston, on behalf of the Galveston Medical Society, delivered an address of welcome couched in eloquent and well-rounded sentences, which did him credit, as well as those he represented.

These warm words of welcome were responded to briefly and in appropriate terms by the President, Dr. Osborn, who then proceeded to business by reading the usual

### PRESIDENT'S MESSAGE AND RECOMMENDATIONS.

*Fellow Members of the Texas State Medical Association:*

To-day our eyes are gladdened by the rich harvest which that little germ, planted twenty-four years ago, in the city of Houston, has borne, and before me are medical men, representing every part of our great State, who are here to demonstrate the great good of this Association, and to participate in the professional and social benefits inuring from that membership. Let us honor that little band of noble workers to whose courage and wisdom we owe all this, and with renewed determination let us continue the good work, remain true to the rich heritage, and fight valiantly to the end.

We believe that under our operations a great reformation ought to be worked out. Time and time again we have attempted, through our learned and diligent committee sent from this body, to get our Legislature to enact a law to regulate the practice of medicine. The failure upon their part should not discourage us. We must keep hammering away at it until quacks are banished from our State, and I would recommend that this Association pass, by a unanimous vote, a resolution requiring its own members to have the moral courage to prevent persons from entering upon the study of medicine whose preliminary education is inadequate for the undertaking. By this, I mean, to use their influence wherever it will avail, and especially in regard to their office students. In this way we can aid the Medical Department of our State University, for they value their examination papers both as to their literary and medical merits; working on this plan, we would soon find our young men eager to take advantages offered them at home, instead of seeking other fountains of knowledge where they are not examined on their literary attainments.

It is absolutely essential for the preservation of the profession, that preliminary examinations upon spelling, grammar, English literature and the classics should be required of any student who may apply for permission to enter upon the study of medicine. After we have taken this broad view and made this stand for higher medical education, and to enable us to make the issue with a rainbow of promise, I would recommend that we, through a committee, petition our legislative powers to give us annually three beneficiary scholarships, to be known as the Texas State Medical Association beneficiary scholarships in the Medical Department of the State University, and the same to be in charge of the three Vice-Presidents of the Association, whose duty it shall be to advertise the same freely, from the the adjournment of the Association until the 15th of September, each year, and the same to be awarded to the student passing the most satisfactory examination in the English branches. Upon a certificate from said officers, the student would receive from the Secretary of this Association his order for a free scholarship. When this is well understood, the medal of the Texas State Medical Association would be eagerly sought after, and I feel confident that one of these three young men would win the prize medal, and it would incite greater interest in this State among the young men entering or intending to enter upon the study of medicine.

If in your wisdom this should seem fair, and you adopt the suggestion, and our wishes and prayers be granted by the powers that be, and I have an abiding faith that they would do so, it would then be right and proper for the nominating committee to elect for Vice-Presidents one from North, Central and South Texas, and have in view those who would take great interest in looking after this important work.

Since the powers that be have established the office of county health officer, without calling on this Association for advice or consultation as to such appointment, it might seem presumptuous or improper in our Association to make them any suggestions, but I can see how we might, through these same health officers, establish a wonderful system of collecting vital statistics; and I would suggest that a committee be appointed at once, whose duty it shall be to explain our wishes to the Legislature, and secure, if possible, an addition to the law governing county health officers, requiring every one of them to keep a correct record of the births and deaths, and endemic and epidemic diseases occurring in the county, and said law to so extend as to compel every physician throughout the State to make weekly reports to the county physician, and the county physician to make monthly reports to the State Health Officer, and the State Health Officer would make annually a report which this Association would have the benefit of. We would then have a reliable record of valuable statistics, and could point with pride to our work, and willingly would our Association crown our State Health Officer with a chaplet of richest jewels.

While we are not permitted by these same powers that be to control or lead in sanitatary affairs, or proclaim quarantine, it does not lessen our duty as an association, nor our duty to our fellow men, to be ever on the outlook, and we must perform our duty faithfully and promptly; we must educate the people in the principles of health and the penalties for their violation. For this we receive no praise or fame, but we still keep the priceless luxury of doing good; to this end I would suggest the appointment or selection of a standing committee, to report annually to the Association on the "sanitary resources of Texas" in reference to its "air, soils and water," both potable and medicinal waters, climate, advantages, and the peculiarities of each particular and peculiar region of the State, with the effects of the same on the people thereof. I know that this work will be arduous, but a committee can make it very interesting, and it will become an important factor in our proceedings.

Another suggestion I would make to you, that we create the office of annual orator, whose duty it shall be to deliver an oration to the Association on the first night after the opening of the session. The election or selection of his subject be left entirely to his choice, and that this office be filled by the nominating committee. We have many gifted and brilliant minds from among your members, and each year we would select one who would interest us greatly, furnishing food for thought and reflection, and thus enable us to pass a pleasant evening. I remember that we once had an essayist selected, but why the custom was dropped my memory fails to recall. Let us try it again, and mark my prediction, the looking forward to that evening's entertainment will be one of the most pleasing thoughts connected with the meeting. It will not only be profitable and interesting, but it will also be on the social order one of the leading features.

I would suggest that a part of the morning of the third day be set aside for the calling of the roll of county medical societies, and hearing reports from them through their accredited delegates. Let us give more prominence to county societies; in that way we can get more influence. Let us extend a cordial invitation to them to send in voluminous reports of their work; thus we get many of the professional brothers who would not otherwise work, interested in our meetings; they begin then to organize more efficiently in the counties, and from the counties they all come to us. Let our worthy Secretary notify each county medical society of a cordial invitation to unite with us. There are a large number of county societies that have never affiliated with us. We should try and get them to attend by their representatives, and thus secure membership of some who might otherwise remain outside. This hour or two devoted to their reports will fill a long felt want to my mind, for I have never yet seen any attention paid to delegates as county representatives. Think over this and let us form a plan to make these visiting delegates feel that their services as delegates are not thrown away. Let us instruct our Secretary to send to each and every county society a cordial invitation to meet with us; send in an annual report of their meetings, their workings, their success, their membership. This interest has been lacking too long; we must have them with us, and have a part of a day set aside for such reports.

In drawing this message to a close, I am pained to inform you that amidst our cordial greetings we have cause for sorrow, as many of our true and tried and honored fellows have passed away, and the roll of dead will show that we too have suffered, and our loss is great yet while we lament them, we feel sure that the good work they did while among us has prepared them to stand unfaltering and undaunted before the great white throne. We will miss them, and never hear Francis, Ward, Pope, Crow, Nicholson and others answer the roll call any more. Peacefully they rest; watched are the mounds over which we weep; loving eyes and fond hearts wait for a reunion with them in the great unknown beyond. I pass this part of the report with bowed head, to the committee on necrology, who I feel satisfied will do all honor to our noble dead, and their report be a credit to them and reflect honor upon the association.

It gives me pleasure to report that no friction has arisen, nor does any now exist between myself and my associates. The government of the association has been an easy matter to handle, for we believe that the government is best which is governed the least. Your able Secretary has done his work well, and I wish to return him thanks, together with my able Vice-Presidents, for their counsel and assistance; and before closing I wish to acknowledge my thanks for the high compliment conferred in making me your presiding officer. To be President of this Association is a distinction worthy of the most vaulting ambition. Now let your generosity extend the mantle of charity over my errors. Believe me, they are ot the head and not of the heart. As we enter upon the business before us, let us each one resolve to make it an harmonious session, replete with good fellowship and full of instruction for our weary brains seeking more light.

Surrounded as we are with so many pleasant environments, I can but augur a happy meeting, and now bid you enter upon the business before you.

The secretary then called the roll, revealing the presence of ninety-one members of the Association.

Reading of the minutes of the last annual meeting was, on motion, dispensed with.

Secretary West read his annual report, which was received and referred to a special committee.

Dr. Loggins then moved that the President's message be referred to a special committee also, which was done.

Treasurer Larendon read his annual report, which was referred to a special committee.

The committee on constitution and by-laws was called on, but not being ready to report was granted further time.

The President's message was referred to a committee of five, who reported later, endorsing the recommendations.

### SECTION WORK.

THE SECTION ON PRACTICE OF MEDICINE was then called. Dr. J. C. Loggins, of Ennis, Chairman, took the chair, and Dr. Clay Johnson, of Corsicana, Secretary.

Dr. Loggins read his address, an able paper on general practice, in which he took a wide range, and discussed State Medicine and Public Hygiene, taking occasion to compliment the public services of State Health Officer Swearingen, and Quarantine Physician Jenkins, of New York. The address was listened to with marked attention and was, on motion, received and referred to the Publishing Committee.*

---

*All the papers and addresses will appear in the annual volume of Transactions.

THE JUDICIAL COUNCIL was called meantime, and Dr. J. C. Loggins, Chairman of Section on Practice, being Secretary of the Council, was relieved by First Vice-President, T. J. Bell, who took the chair and presided till the close of the Section. Vacancies were filled by the President, and the Council, as organized, was composed as follows:

P. C. Coleman, M. D., President, Colorado.
J. C. Loggins, M. D., Secretary, Ennis.
S. R. Burroughs, M. D., Raymond.
E. L. Menefee, M. D., Granbury.
A. B. Gardner, M. D., Belleville.
H. H. Darr, M. D., Caldwell.
Irwin Pope, M. D., Tyler.
A. N. Denton, M. D., Austin.
R. E. Moss, M. D., San Antonio.
W. P. Powell, M. D., Willis.
W. L. York, M. D., Decatur.
J. D. Burch, M D., Aurora.

They retired for work and Section of Practice was resumed.

Dr. H. A. West read a paper on "The Association of Deafness and Morbid Processes."

Prof. Allen J. Smith, of the Medical Department Texas State University, opened the discussion on Dr. West's paper. He agreed thoroughly with the views held by Dr. West. Single lesions were the exceptions rather than the rule. In a vast number of post-mortem examinations he had found this to be the case, and had found a complicity of diseases. In typhoid fever, for instance, the system is weakened, and the disease attacks other organs of the body any weak spot in the system would surely suffer. He cited a case of pneumonia, where, after death, lesions of the heart, kidney, etc., were found. He then showed why it was perfectly natural that such conditions should prevail and spoke of the diseased liver, and pointed out how the flow of the blood was impeded, the circulation made sluggish, and how these conditions affected all the organs in the human body. His address was extremely interesting and instructive, and was listened to with rapt attention. He closed with the statement that in his experience a complexity of lesions is found in post-mortems rather than single ones.

Further discussion was invited by Chairman Bell, but no one cared to speak.

On motion, Dr. West's paper was received and referred to the Publishing Committee.

The next paper was by Dr. David Cerna, of the Galveston Medical College, on "The Action and Uses of Pental." Dr. Cerna first explained what pental is, and then read an exhaustive report of experiments made by himself and others, showing the effects of the drug on the heart, nerves and circulatory system. The paper was very complete and gave evidence of thorough investigation and deep thought on the part of Dr. Cerna. At the conclusion of the reading a vote of thanks was given Dr. Cerna for his valuable contribution. Referred to the Publishing Committee.

Prof. Thompson, of the Texas Medical College, discussed the paper. In his opinion the drug was a chemical curiosity, nothing more. As a practical remedy it was useless if not absolutely dangerous. He told of tests made by Dr. Cerna and himself in the surgical wards of the hospital. In two cases it was given a fair trial and in neither was the patient under the influence of the so-called anesthetic and in both recourse had to be had to chloroform. The Doctor created a laugh by telling how one of the patients kicked, literally kicked, when they tried to perform a minor surgical operation on him after giving him a dose of pental. "In my opinion," said he, in conclusion, "the drug is useless as a remedy, is dangerous and has the most disgusting smell one ever came in contact with. The whole ward was filled with its disgusting odor when we used it."

The next paper was by Dr. C. H. Wilkinson, of Galveston, on "Concussion of the Spine," in which he treated at length those special cases due to railway accidents. His conclusions were that many of the so-called cases were the result of fright and shock to the nervous system rather than to any actual injury received. He did not question that the patients suffered, but drew attention to the fact that many of the worst cases quickly recover so soon as they receive damages from the railways for the injury done them. Such cases could not have been those where actual injury had been done the spinal cord, and yet such patients have just as genuine symptoms of concussions as the others.

Dr. Wilkinson's paper was received and a vote of thanks extended the doctor.

Several members asked that some of the railway surgeons present discuss the paper, as in their opinion it was too valuable not to have further notice taken of it at this meeting. None of them spoke, however.

Prof. Allen J. Smith thought that Dr. Wilkinson included too many of the immediate symptoms of accidents and in his opinion some of the subjective symptoms were of great importance. The fact cited by Dr. Wilkinson that some of the patients were cured by receiving money from the railroad proved nothing except that it made the conditions of a speedy recovery more favorable. Every physician knows that peace of mind and absence of worry are favorable conditions for recovery. Money is often the means of prolonging life if not of saving it. The rich consumptive will live longer than the poor one. The cases cited by Dr. Wilkinson were just those which would be most readily affected as they were due to mental and nervous causes.

Dr. Kennedy, of San Antonio, attempted to tell of two cases of "railroad spine" which had come under his observation, but was cut off by Chairman Bell who stated that the limit of time for discussion had expired.

"Continued Fevers of Texas Classified" was the title of the next paper, read by Dr. B. F. Brittain, of Baird.

He placed all Texas continued fevers in two classes, typhoid and malarial, and then showed that these two were often so much alike that the physician could not tell them apart. "I've made the mistake," said he, "and I see by the way you fellows dodge and look at each other that you have all made the mistake of treating malarial fever for typhoid fever, and vice versa."

Dr. West was much pleased with "the hard, common sense paper" of Dr. Brittain, and he thanked him for it.

Dr. O'Barr agreed with Dr. Brittain, and mentioned several cases which had come under his own observation, going to show the correctness of Dr. Brittain's views and conclusions.

Dr. Sears, of Waco,—the President elect for 1893-4, was skeptical. "I admit," said he, "that there is a great deal of typhoid fever in Texas, and perhaps more of it in Galveston than elsewhere, as Dr. West seems to claim. Perhaps we have typhoid fever up our way, for we have had considerable continued fever there until lately, when we got good artesian water. The change in water may have changed the disease, for they claim that typhoid fever is caused by drinking water in which the little typhoid bugs live.* They get into the system through drink-

*Dr. Sears is not a believer in the germ theory of disease, and calls microbes, "bugs."—Ed.

ing water, though if they have legs and wings and can fly about, I don't see why they have to get into the water before they can get into the human body. They should be able to fly in as well as to float in. But that has nothing to do with it. We have had considerable, and still have some continued fever in Waco. Perhaps it is typhoid, as Dr. West claims, but if it is, then he will have to admit that quinine will cure typhoid fever, for that is all we give our patients.''

Dr. West: ''How long does it take to cure with quinine?"

Dr. Sears: ''From one to two and sometimes three weeks.''

Dr West: ''Do you call that curing?''

Dr. Sears. ''Yes; don't you?"

Dr. West: "No, sir; I call it surviving the treatment."

Dr. Sears then proceeded and described the symptoms and treatment. The cause was malaria, and the remedy quinine. He spoke of hundreds of cases of continued fever, all of which had been treated with quinine, and all which had recovered.

Dr. Pope asked Drs. West and Brittain if they denied the existence of catarrhal fevers in Texas. He could not see why the mucous membranes of the intestines should be exempt from fevers any more than the mucous membranes of the eye, throat or ear.

Dr. West answered that he admitted that we had catarrhal fevers to a limited extent, but they were not so prevalent as popularly supposed, and that mild forms of typhoid fever were often mistaken for them.

Dr. Shenck spoke of temperature charts of typhoid fever, and stated that if the cases spoken of by Dr. Brittain were typhoid, then these charts would have to be changed.

The hour for adjournment having arrived, an attempt was made to continue the discussion of Dr. Brittain's paper over until morning.

Chairman Bell stated that there was another paper on the same subject, which would admit of a full and free discussion.

On motion, Dr. Brittain's paper was referred to the Publishing Committee and the Association adjourned until 9 o'clock sharp Wednesday morning.

---

### SECOND DAY, WEDNESDAY May 3.

### MORNING SESSION.

At 9:30 the meeting was called to order. President Osborn in the chair. Prayer by Dr. Carter, of Grace (Episcopal) church.

Reading of minutes of yesterday dispensed with.

The Committee on President's recommendations reported, endorsing the same. Later, in pursuance of their recommendation a committee composed of Dr. J. W. McLaughlin, Dr. F. E. Daniel, Dr. A. N. Denton, Dr. C. M. Rosser, Dr. G. W. Christian was appointed to go at once to Austin and lay the matter before the Legislature, then in session, and endeavor to secure the passage of an act in accordance with the President's views.*

The COMMITTEE ON REVISION OF CONSTITUTION, appointed at Tyler meeting, was called on for report.

They submitted two reports; one by Secretary West, and one by Dr. Sears, the only members of committee present, differing in some essential features.

Both reports were read by Secretary West. He explained the differences, which related to the attitude of the State Association towards County and District Associations and the rules governing trials and discipline. Drs. Sears insisted on making membership in a County or District Association a pre-requisite to membership in the State Association, while he (Dr. West), insisted that such a rule would be unfair, and would exclude from the Association many good and desirable physicians, who for one cause or another, might not care to become members of their local organization.

Dr. Sears argued in favor of his position. In his opinion, it was the duty of the State Association to foster and build up County and District Associations, and nothing would do this so well as the rule he advocated.

Dr. Clark favored both reports in part, and neither in its entirety.

Dr. Christian indorsed Dr. West's position, and considered it the wisest plan for the Association to adopt.

Dr. Brittain spoke in favor of county organizations being given the greatest encouragement, and personally he was in favor of doing everything possible to foster them. "They are great institution," said he. "The other day they organized the East Texas Medical Association, and they had the good sense to elect me President. That settles it. You can stop right now."

---

* [This committee went to Austin on adjournment of State Association (Saturday May 6), but found that the Legislature was about to adjourn. They therefore organized and adjourned to meet at some future day and bring the matter before the next session of the Legislature.—ED.]

Prof. Allen J. Smith thought that the difficulty could be gotten over by allowing non-members of County and District Associations to join the State Association, provided no objection is filed against them from members of such Associations.

After some further discussion a vote was taken, and it was decided to reject the amendment of Dr. Sears and leave the question just as it was.

The 1st. Section of the Constitution was then read as follows: "Every regularly educated man within the limits of the State, who is a graduate of a regular medical college in good standing, and who adopts and conforms to the code of ethics of the American Medical Association, shall be eligible to membership in this body."

The reading of this section precipitated another discussion. Dr. Rosser moved that the word "white" be inserted before "man."

Dr. Knox requested him to explain what he meant by "white," and wanted to know whom he was anxious to exclude.

Dr. Rosser said it was well known what "white" meant, and that it was the intention to exclude negroes.

Professor Keiller: "I have seen much of the civilized world and yet it has remained for me to come down here in Texas to find that a man of scientific education and training should be excluded from a body composed of scientific men because of his color."

Professor Clopton: "The gentleman has not been in the South long enough to appreciate the prejudice which exists in the minds of the Southern people against anything like social equality between the whites and negroes. I desire to offer an amendment to that section, which is that every regularly educated physician, except negro physicians, shall be eligible to membership in this body."

This amendment was seconded and carried by an overwhelming majority. It permits lady physicians who are graduates of regular colleges, and who are otherwise qualified, to become members of the Association.

Dr. C. L. Gwyn, of Galveston, offered an amendment, having for its object the election of officers of the Association by all the members present, and doing away with the nominating committee.

After considerable discussion, a vote was taken, and Dr. Gwyn's amendment was rejected.

The question of trials and discipline having been practically settled by the continuation of the rules defining the attitudes of the State Association to County and District Associations, no great discussion was had, and the by-laws formed by Dr. West was adopted.

A vote was then taken on the constitution and by-laws as a whole, and it was adopted.

The Committee on Treasurer's Report found it correct and endorsed same.

Dr. West stated that a medical journal was anxious to secure the paper read by Dr. David Cerna on Tuesday, and moved that the rule prohibiting the publication of papers read before the Association previous to their appearance in the published proceedings be suspended in the case of Dr. Cerna. This gave rise to a lively discussion, quite a number of members standing out for the enforcement of the rule. Dr. Cerna explained that he was anxious to secure the publication of his paper at the earliest date possible, since he had embodied in it the results of scientific investigations made by himself, and he was anxious to secure priority for them. If he waited until the regular proceedings were published, some one else might make the discoveries he had made, and he would lose all the credit and honor.

Dr. Scott moved that any member of the Association may be permitted to publish his paper or papers at any time he sees fit to do so, provided due credit is given the Texas State Medical Association.

A motion to table this was lost, and then it was carried by a good majority.

At this point the Judicial Council came in, and reported the following named gentlemen entitled to membership:

Dr. John B. Haden, Galveston.
Dr. J. E. Wall, Carthage.
Dr. T. Flavin, Galveston.
Dr. Allen J. Smith, Galveston.
Dr. A. H. Norton, New Braunfels.
Dr. F. M. Duke, Alvin.
Dr. R. M. Sproule, Wallisville.
Dr. C. C. Barrell, Galveston.
Dr. W. J. Bearers, Houston county.
Dr. J. M. Largent, McKinney.
Dr. J. W. Hale, Waco.
Dr. W. A. McAlpine, Galveston.
Dr. V. P. Armstrong, Dallas.
Dr. A. J. Farrell, Wharton.

The resignation of Dr. J. M. Litten, of Austin, was read and referred to the Judiciary Council. Accepted, and Dr. Litten was then elected an honorary member.

On motion, Dr. S. O. Young, of the *Galveston News*, formerly Secretary of the State Medical Association, was elected an honorary member of the Association.

The hour for adjournment having arrived, the Association adjourned until 2:30 p. m.

AFTERNOON SESSION.

Section on Practice resumed work, First Vice-President Bell presiding.

The first paper was one by Dr. James Kennedy, of San Antonio, on "Reconversion of Peptone into Albumen, etc."

Dr. Kennedy explained that he had not had time to complete his paper so as to show the medical portion of it, but would read what he had, which related to chemical changes he had found to take place. He then explained a number of investigations he had carried on, and gave the results. The paper was very learned, but dealt too much in chemical changes, etc., to prove very attractive to the average physician.

Dr. Cerna discussed the paper. He said that Dr. Kennedy had gone over the ground very thoroughly, and had conducted his investigation with much intelligence. There were many factors which enter into the coagulation of the blood. Just what the important one is, we are unable to say. It is true that we have many theories, but we know few absolute facts. Dr. Kennedy had spoken of fibrine plastine and the difficulty of separating it, and yet it was not necessary. The same is true of fibrinogen and fibrine ferment. Both may be present, and yet no coagulation occurs until an inorganic salt is introduced. In his opinion, an inorganic salt is the chief factor in coagulation of the blood. The subject was one inviting the closest study and investigation, and he hoped Dr. Kennedy would continue his research.

On motion, Dr Kennedy's paper was referred to the Pnblishing Committee.

There were other papers on general medicine, but the time having expired they were all referred to the Publishing Committee, and the

SECTION ON OBSTETRICS AND DISEASES OF CHILDREN

was called on.

Dr. Irvin Pope, of Tyler, took the chair, and Dr. I. E. Clark, of Schulenburg, acted as Secretary.

Dr. Pope read the report of the chairman. It covered a great deal of ground, and dealt with subjects with which all present were perfectly familiar, and with which they had had much experience. The first portion dealt with obstetrics, and the second with diphtheria and kindred diseases of children.

Dr. Sears spoke of that portion relating to membranous sore throat, and gave his experience in its treatment. He was listened to with great attention, for all present value his extensive experience.

Dr. W. R. Blaylock, of McGregor, referred briefly to the obstetrical, and then passed to other portions of the paper, to diphtheria and membranous croup. In his opinion, there were two kinds of diphtheria bacillus, only one was the genuine, and when this was present the patient generally died. He related a recent case in his practice, that of a child. All the symptoms were those of true diphtheria, and yet no other cases followed, though the conditions were very favorable for their development. Just when he got ready to perform tracheotomy, the child died. He performed the operation anyway.

Prof. Thompson referred to the obstetrical portion of the report, and spoke of the treatment of the cord in newly born infants. Dr. Pope had stated that the cord required no treatment or special bandages. Prof. Thompson differed with him, and thought that in this climate the cord should be treated on surgical principles, and should be washed with antiseptic preparations. There was so much in Dr. Pope's paper that he scarcely knew where to begin or end a discussion of it. He would content himself by advocating the use of antiseptics on the cord.

Dr. Christian settled the question of antiseptic treatment—at least to his own satisfaction—by stating that he had never tried it but once, and that two weeks later he was sent for by the mother, who wanted to know what on earth he had done to that cord, for it was as fresh and green then as when he had dressed it. That cured him of using antiseptics.

This closed the discussion of Dr. Pope's paper, and the next paper was read. This was on "Paracentisis Capitis in Hydrocephalus," the report of a case by Dr. N. A. Olive, of Waco.

Prof. Thompson opened the discussion. He thought that frequent tapping in such cases was useless, if not absolutely harmful. Some method of drainage would be better. The prolongation of the life of a child for a week or two was absolutely useless, as death was inevitable. He could see no good that could come from it.

Dr. Olive's paper was then referred to the Publishing Committee.

"Placenta Prævia with Instruments for Treating Same," was the title of the next paper, read by Dr. Q. C. Smith, of Austin.

Prof. J. F. Y. Paine, of Galveston, discussed this paper at some length, and delivered an admirable address on the subject, pointing out that the chief danger from placenta prævia is hemorrhage, and suggesting the best modes for preventing and arresting this.

After some further discussion by Prof. Keiller, and a rejoinder by Dr. Smith, the paper was referred to the Printing Committee, and the Section on Obstetrics adjourned.

SECTION ON SURGERY.

The Section on Surgery was then opened, the chairman, Dr. A. B. Gardner, of Bellville, postponing his report until morning, in order to allow Prof. J. E. Thompson an opportunity to read a paper on "Whitehead's Operation for Hemorrhoids Considered from an Anatomical and Pathological Standpoint." Prof. Thompson had several anatomical specimens and plates with which he illustrated his lecture. The paper created great interest.

Dr. A. F. Sampson, of Galveston, having a paper on the same subject, was invited to read it, so that both might be discussed. He did so, delivering a fine address on "A Modified Whitehead's Operation." The modifications were his own, and were intended to do away with the chief objection to Whitehead's operation, the danger of hemorrhage.

The discussion was full, and covered the ground thoroughly. It was opened by Dr. Hadra, and participated in by Dr. Brittain, Dr. Knox, and others. The discussion was extremely interesting.

Adjourned till 9 a. m., Thursday.

---

### THIRD DAY.

MORNING SESSION.

Prayer by Rev. Dr. Byrd, Episcopal church.

Minutes dispensed with.

SECTION ON SURGERY

resumed, Chairman A. B. Gardner in the chair.

Dr. Gardner, as chairman, read his report, which had been passed over the previous evening, in order to allow Prof. Thompson to read his paper. Dr. Gardner's report was listened to with rapt attention, and at the conclusion of the reading was referred to the Publication Committee, without debate, the reports of chairmen not being debatable.

"A Case of Splenectomy, with Recovery," was the title of the next paper, by Prof. J. F. Y. Paine, of Galveston, and proved a most interesting one. It was a report of a case in which the spleen had been successfully removed from a lady in this city by Dr. Paine. The paper described at length the history of the case, and gave each detail of the operation, and the treatment afterward. The lady herself was present, and her appearance was the best evidence of the success of the operation. The paper was received, and referred to the Publishing Committee.

Dr. Clay Johnson asked if the blood of the lady had been examined since the operation, and was told that no satisfactory examination had yet been made, because of the want of proper instruments at the hospital with which to make it.

Dr. J. D. Osborn asked if the prognosis was such that Dr. Paine thought that the woman would die if the operation were not performed. He asked because he had had a similar case in his own practice recently, one which presented nearly the same symptoms that appeared in Dr. Paine's patient, and which had recovered without an operation.

Dr. Pope described a case where an operation was indicated. The cause in this was an injury received in a railroad wreck. The patient received $5000 damages, and was enabled to change climates, and the last time he heard from him, he was well.

Dr. Shearer, of Wallisville, gave the history of an interesting case, recently occurring in his practice.

The next paper was called for. "The Treatment of Depressed Fractures of the Skull," was the title of an interesting paper by Dr. P. C. Coleman, of Colorado. It was received and referred.

Dr. B. E. Hadra, of Galveston, was much pleased with Dr. Coleman's excellent paper. In his opinion, it was a superb report, and so rich in suggestive facts that it was hard to find the most appropriate point to discuss. However, he would say that in all cases of the kind reported, he favored the most thorough exploration of the brain. "You need not be afraid," said he, "it is utterly ridiculous the way you can probe and cut into some parts of the brain. When the injury is on the forehead, or the front part of the brain, you can even cut pieces of the brain away, and it will do no harm. I've tried it, and am simply telling you what I know from experience. I don't know what use the front part of the brain is any way." He then related a number of cases in which he had cut huge chunks of the skull out, and run his finger around the convolutions of the brain, seeking for injured portions. In only one of perhaps twenty such cases, had the patient died, and that was a long time after the operation had been performed. Before Dr. Hadra had finished, his time had expired, but by unanimous consent of the members, he was permitted to continue his interesting talk.

Dr. C. A. Smith, of Tyler, told of a case which had recently come under his treatment. The man's skull had been literally torn off on one side of his head, and a deep furrow cut in the brain substance, by a railroad engine. The man recovered, and is now working on a section. In his opinion, immense injury could be done the brain without apparent bad effect.

The Section on Surgery was then suspended, the Judicial Council came in, and reported the following gentlemen eligible to membership, and they were elected members:

Dr. L. C. Red, Houston.
Dr. C. T. Hughes, Houston.
Dr. J. W. Scott, Houston.
Dr. W. J. Ducie, Galveston.
Dr. Frank King, Lampasas.

MEMORIAL SERVICES.

The hour set aside for holding memorial service having arrived, President Osborn called Dr. C. M. Rosser, of Dallas, chairman of the Committee on Necrology, to the chair.

Dr. Rosser stated that Dr. E. J. Ward, of Waxahachie, was the only active member of the Association who had died since the last meeting of the Association. He then reviewed the life and works of Dr. Ward, and paid high and feeling tribute to his memory. It was suggested that Dr. A. B. Burroughs, a brilliant young physician, had also died during the year. He not being an active member at the time of his death, however, no official notice could be taken of it, according to the rules of the Association.

It was then moved and carried that all ex-members of the Association who had died during the year be placed on the roll of active members, and that their names be enrolled among the list of active members who had died. A committee was appointed to draft suitable resolutions, to be presented at the meeting in the morning.

Adjourned.

AFTERNOON SESSION.

Chairman Gardner called the Association to order promptly at 3:30 o'clock, and the sitting of the

SECTION ON SURGERY

was resumed.

"The Treatment of Tetanus," was the title of a splendid paper by Dr. George H. Lee, of Galveston. It was received, and referred to the Publishing Committee.

The members were anxious to discuss Dr. Lee's able paper, and one or two tried to do so, but the chairman stated that there were too many papers yet to be read, and too many Sections to report, to allow discussion. All the other papers were then read by caption, and referred to the Publishing Committee.

This closed the work of the Section on Surgery, and the Section stood adjourned.

SECTION ON MEDICAL JURISPRUDENCE

was called on. The Chairman and Secretary both being absent, and no papers being before the Association, that Section was passed.

SECTION ON STATE MEDICINE

was then convened, Dr. C. M. Rosser, of Dallas, in the chair, and Dr. I. E. Clark, of Schulenburg, Secretary.

Chairman Rosser then read a valuable and interesting paper on

PRACTICAL METHODS OF SEWAGE DISPOSAL.*

Dr. Blaylock said he had listened with the greatest interest to Dr. Rosser's magnificent paper, and was much impressed by it. It was of the greatest importance to every town and city. The plan of subjecting fæcal matter to the high temberature of 350 degrees destroyed all disease germs, and rendered it harmless.

Dr. Armstrong, City Physician of Dallas, spoke in high terms of the apparatus of Dr. Rosser. It was the most perfect system he knew of.

Dr. Burroughs, of Houston, indorsed the paper from the first line to the last. The apparatus described by Dr. Rosser was the great need of the hour.

Dr. Christian thought the most important duty a physician

---

*This paper will appear in full in the Texas Sanitarian for May.—Ed.

has to perform, is to protect the public health. No small towns relying on wells for a water supply could be healthy, unless the proper removal of filth was looked after. The physician should impress on the people the importance of proper sewers and the disposal of sewage. He regarded a system of disposing of sewage as cheaply as the one offered by Dr. Rosser, a public blessing.

Dr. Brittain gave Dr. Rosser's apparatus the strongest indorsement. "It is to our interest to let the people get sick," said he, but we don't want them to get too sick. I regard the paper as the best and most timely that has yet been read, and think the people of Texas should have the benefit of it at once. I therefore move that our rule be suspended, and that the paper be given to the press of the State, with the request that they publish it and give it the widest circulation possible."

This was seconded by half a dozen members, and it was carried unanimously.

Dr. Burroughs, of Houston, suggested that the newspapers be requested to head the article "Cholera" in big letters.

Another member suggested "C. C. C." as a suitable heading, stating that these letters stood for "Certain Cholera Cure."

Dr. David Cerna was to have read a paper, "Some Thoughts on Higher Medical Education and Medical Ethics,"* but suggested that in view of the many important papers yet to be read his own be referred to the Publishing Committee, which was done. The members took this action with regret, for recognizing Dr. Cerna's ability they looked forward with pleasant anticipation to the reading of his paper. The

SECTION ON GYNECOLOGY

was then called, Dr. J. F. Y. Paine, Chairman, and Dr. George H. Lee, Secretary.

Dr. Paine delivered an admirable address. It was thoroughly scientific, as all that comes from his pen is; it was replete with common sense suggestion and in this respect intensely practical. Every mother and every keeper of a boarding school for young ladies should have a copy of the first portion of it. Unfortunately the rules of the Association prohibit the discussion of a chairman's report, but, fortunately they do not prohibit thinking about it, and that is what most of the members will do for some time about Dr. Paine's paper. Dr. Paine, being called away at

---

*Dr. Cerna's paper on Medical Education and Medical Ethics will appear in next number of DANIEL'S TEXAS MEDICAL JOURNAL.—ED.

the conclusion of his address, requested Dr. Osborne to preside over the Seetion.

"A Contribution to the Pathology of the Fourchette" was the title of an interesting paper read by Dr. B. E. Hadra, which was referred to the Publishing Committee.

"Cervical and Corporeal Endometritis" was the next paper read by Dr. Wm. M. Cunningham, of Bastrop. Received and referred.

All the other papers were read by caption and referred.

"Pelvic Peritonitis and Cellulitis" was the title of a paper to have been read by Dr. Wm. Keiller. He wished to withdraw it, and after some discussion was permitted to do so. The Section was then adjourned.

Dr. C. F. Paine, a member of the Committee on President's Message, asked that the committee report, made during the morning session, be returned to the committee, so that the report could be amended. The request was complied with.

The Nominating Committee, of one member from each county, then came in and submitted the following list of officers and committee-men for the ensuing year:

NEW OFFICERS.

President, J. H. Sears, Waco.
First Vice-President, C. M. Rosser, Dallas.
Second Vice-President, E. M. Rabb, Hallettsville.
Third Vice-President, W. A. Watkins, Kemp.
Secretary, H. A. West, Galveston.*
Treasurer, J. Larendon, Houston.*

JUDICIAL COUNCIL.

To fill vacancies on the Judicial Council: R. Rutherford, Harris; S. Cunningham, W. B. McKnight, Parker; L. Ashton, Dallas. Orator, W. R. Blaylock.

Section on Practice of Medicine: J. C. Jones, Chairman; I. E. Jones, Secretary.

Section on Obstetrics and Diseases of Women: Ed. Randall, Galveston, Chairman; A. B. Gardner, Denison, Secretary.

Section on Surgery: C. A. Smith, Tyler, Chairman; Clay Johnson, Corsicana, Secretary.

Section on Medical Jurisprudence and Psychology: B. F. Brittain, Jacksonville, Chairman; C. F. Paine, Comanche, Secretary.

---

*Elected in 1891 for five years; holds over.—Ed.

Section on State Medicine and Hygiene: T. J. Bennett, of Austin, Chairman; A. M. Armstrong, of Crawford, Secretary.

Section on Gynecology: G. W. Christian, Burnet, Chairman; R. W. Knox, Houston, Secretary.

Section of Ophthalmology and Otology: J. V. Spring, of San Antonio, Chairman; ——, Secretary.

Section on Dermatology: F. E. Daniel, Austin, Chairman; S. E. Hudson, Austin, Secretary.

Committee on Publication: Continued.

Committee on Necrology: Continued.

Committee on Microscopy and Pathology: Allen J. Smith, Galveston.

Committee on State Board of Health: R. M. Swearingen, Austin.

Time of holding next meeting: Fourth Tuesday in April, 1894. Place: Austin, Texas.

Committee on Arrangements: A. N. Denton, Austin.

Adjourned to Friday morning.

### FOURTH DAY—FRIDAY.

### MORNING SESSION, MAY 5, 9:45.

No minister present, prayer dispensed with.

Section work was resumed, and Dr. C. F. Paine, of Comanche, read an exhaustive and valuable paper entitled "Pneumonitis," some notes on its treatment, which was received and referred to Publishing Committee.

Dr. David Cerna was much pleased with Dr. Paine's excellent paper and thought that it was worthy of the freest and widest discussion. There was one fact, which, in his opinion, should never be lost sight of—in pneumonia the disease is in the lungs, but the danger lies in the heart. He, therefore, considered cardiac stimulants as of the utmost importance. Alcohol might be used in the first stages, but never in the latter, because alcohol was a depressor as well as a stimulant and the constant use of it during the progress of the disease would necessitate larger doses later on, which might have the reverse effect of that intended. He warned the members against the use of coal tar derivatives. They were all, without exception, heart depressors. The use of antipyrine in pneumonia he considered as absolutely criminal. In the latter stages of pneumonia the heart was weak from overwork and anything that depressed it, might prove fatal. He favored the use of digitalis which possessed the quality of being a stimulant as well as furnishing food for the heart. Digitalis feeds the heart and stimulates the nerve centres. He favored using it in large doses; never in small ones.

Dr. Knox, of Houston, said that pneumonia was a self-limiting disease, and in his opinion the least medicine given the better it was for the patient,

Dr. Christian thought that the duty of the physician in such cases was first to relieve pain and then insure the recovery of his patient. Counter-irritants would do the former and proper food the latter. He then related some recent cases in his own practice, when the free use of cold water had been followed by the happiest results.

Dr. S. Ashton, of Dallas, endorsed the cold water treatment and also advocated blood letting. He knew that bleeding was not fashionable or popular but he had tried it of late, and in every case where it was indicated and he had bled, the best results were obtained. He had tried it time and again.

Dr. Cunningham also advocated cold water in pneumonia but thought extreme caution should be observed in its use. The sudden application of cold water often caused more injury than good. This was an important point for the physicians to bear in mind.

Dr. H. A. West, at the request of Dr. Cerna, discussed the paper and gave it the strongest indorsement.

Drs. Denton, Shearer, McLaughlin and Bell also made interesting talks, giving their conclusions drawn from experience as to the best methods of treating the disease uuder discussion.

The Executive Session was then resumed and the minutes of whole session were read and, after corrections had been made in some points, they were approved.

The remaining Sections were called on and all papers were read by caption and referred to the Publishing Committee.

The Section on Ophthalmology and Otology held a special session in the ladies' waiting room of Harmony hall, Dr. George P. Hall, Chairman, and Dr. William H. Baldinger, Secretary.

Promptly at 2:30 o'clock p. m., the Section was called to order.

In lieu of the usual annual report on the progress in ophthalmology and otology, Dr. Hall read a very interesting paper, entitled, "A Contribution to the Study of Insufficiencies of Ocular Muscles and Measures Directed to Their Relief," which was favorably received and fully discussed by Dr. Pope, of Dallas, Dr. Hodges, of Galveston, and Dr. Moss, of San Antonio.

The next paper on the list, "Trachoma or Granular Conjunctivitis," by Dr. Haughton, of Midland, was read by caption and referred to the Publishing Committee, as was the paper by Dr. Carter, of Corsicana, entitled "Paralysis of Accommodation as a Sequelæ."

Dr. Spring, of San Antonio, next read an interesting paper entitled "Neurosis of the Eye Due to Stricture in the Male Urethra, With Cases," which was discussed at length by Dr. Chilton, of Dallas, Dr. Hall and Dr. Hodges, of Galveston, and Dr. Irvin Pope, of Tyler.

Dr. B. A. Pope, of Dallas, next related several interesting cases of tobacco amblyopia, which were instructive from the fact of treatment by hypodermatics of cocaine and combined medical tion of pilocarpine, cocaine and strychnine, and also entertained the Section with experiences with menthyl-violet in carbuncle, furuncle and "treatment of certain tumors of the face and eyelids with menthyl-violet."

Dr. Hodge's paper on "Dislocation of the Lens" was then read and fully discussed by Drs. Pope, Spring, Chilton and Hall.

"Treatment of Strabismus," by Dr. Chilton, of Dallas, proved an interesting paper, and was discussed by Drs. Moss, Haden, Spring, Pope, Hall and Hilgartner.

A paper by Dr. Hilgartner, of Austin, entitled "Thiersch's Skin Grafts for Pterygium," proved most interesting and was most favorably received, and there being no further business the Section adjourned.

In an informal meeting after adjournment, the question as to the advisability of forming

A STATE OPHTHALMOLOGICAL AND OTOLOGICAL SOCIETY

was discussed, and a committee of three—Drs. Hodges, of Galveston, Spring, of San Antonio, and Hilgartner, of Austin—was appointed to take active steps during the coming year to formulate matters concerning the organization of such a society, and to correspond with all ophthalmologists in the State, and report at the next meeting of the State Medical Association.

Dr. Hall announced that the formation of this society was in no manner to detract from the membership of the State Medical Society, but simply intended to bring all interested in this field to closer relation and fellowship. It is the intention of the organizers to meet annually at the same point as the State Association, and one day previous, and will in no way interfere with the work of the Association, but to develop more strength and interest in section work of ophthalmology and otology.

THE PRESIDENT'S ADDRESS

Was delivered at 8 p. m., Thursday, third day, at the Garten Verein: after which a dance was indulged in by the young people, a great many ladies being present. A splendid "buffet lunch" was spread in the Club house at the "Garten," and members indulged in "bowling," both at the alley and at the Club house. The social features of the meeting were very pleasant. A reception at the house of Congressman-elect Gresham; a ride over the city, several sails on the bay and excursions to the jetties. On Friday a large number of delegates and citizens with their ladies made a trip on the U. S. Revenue Cutter Galveston, down and all around the bay; another party on the quarantine steamer Hygeia, etc., etc. There was no banquet and no speeches.

There were four of the original organizers of the Association on the floor; and another, who as a medical student, participated in the birth of the Association—Dr. D. F. Stuart, the only man now living who signed the original call for the first meeting in Houston; Dr. T. J. Heard, of Galveston, the first President of the Association; Dr. J. H. Sears, of Waco, who had just been elected President; Dr. J. Larendon, the perpetual Treasurer, who has never missed a meeting since the organization.*

Meantime, in general session the President appointed the following Committee on Scholarships in the medical college: Drs. J. F. Y. Paine, H. A. West and Ed Randall.

Dr. Tucker introduced the following resolution, which was adopted:

*Resolved*, that it is in violation of the code of ethics for a member of this Association to associate himself in partnership with an undergraduate, and that any violation of this will be punished by expulsion from this Association.

Dr. Christian moved—and it was duly seconded and carried—that a special vote of thanks be given Dr. T. Flavin for the excellent stenographic record of the meeting he made.

Dr. Allen J. Smith spoke of the efforts being made by the medical college to secure specimens for the college museum, and invited all members to contribute specimens.

The hour having arrived for the induction of the new officers of the Association into office, President Osborn appointed Dr. D.

* Dr. D. R. Wallace, of Waco, was one of the organizers we are quite sure.—ED.

F. Stuart, one of the founders of the Association, and Dr. P. C. Coleman as a committee to escort President-elect Sears to the stage.

The committee performed its duty gracefully, both Drs. Stuart and Coleman making happy speeches in introducing the new President. Dr. Sears returned his thanks for the honor, saying that he rather regretted that the honor had been put upon him, for it deprived him of the greater distinction of being the only one of the organizers of the Association who had never held office. His speech was warmly applauded.

Drs. Bell and Grammer were appointed by the chairman to conduct Vice-President Rosser to the stage. Dr. Bell introduced Vice-President Rosser in a neat speech, and Dr. Rosser thanked the Association for the honor done him.

The second and third Vice-Presidents were absent, and therefore could not be inducted into office.

Dr. Osborn, the retiring President, made an eloquent and feeling address in turning over the high office he had held to his successor. He thanked the Association for the honor it had conferred upon him, and the members for their uniform courtesy and the assistance they had given him.

At the conclusion of Dr. Osborn's address Dr. Bell offered a series of resolutioms, which were adopted, returning thanks to the railroads, the mayor and citizens of Galveston, Colonel and Mrs. Gresham, the *News* and the *Post* for their reports, etc.

Resolutions were also adopted indorsing in the strongest terms the John Sealy training school for nurses.

On motion of Dr. J. F. Y. Paine, a vote of thanks was given Dr. Orborn, the retiring President, for the able and impartial manner in which he had conducted and managed the affairs of the Texas State Medical Association during the past year.

The Association then adjourned to meet next year in Austin.

---

Circulars are being scattered among the physicians of the United States in favor of the proposed international memorial to be erected to the honor of Semmelweis, whose great service in establishing our knowledge of the origin and prevention of puerperal fever and the antiseptic treatment of child-bearing women is now generally recognized. Subscription can be forwarded by P. O. or by check to the Treasurer, Dr. J. Elischer, Budapest, Hungary, Petöfi-tér No. 1.

